# Intradermal vaccination with a phytoglycogen nanoparticle and STING agonist induces cytotoxic T lymphocyte-mediated antitumor immunity

**DOI:** 10.1038/s41541-024-00943-8

**Published:** 2024-08-17

**Authors:** Juan F. Hernandez-Franco, Imran M. Jan, Bennett D. Elzey, Harm HogenEsch

**Affiliations:** 1 Department of Comparative Pathobiology, College of Veterinary Medicine, Purdue University, 725 Harrison Street, West Lafayette, IN 47907 USA; 2 Purdue Center for Cancer Research, Purdue University, West Lafayette, IN 47907 USA; 3 Purdue Institute of Inflammation, Immunology and Infectious Disease, Purdue University, 625 Harrison Street, West Lafayette, IN 47907 USA; 4 Present Address: Department of Developmental and Molecular Biology, Albert Einstein College of Medicine, 1225 Morris Park Ave, Bronx, NY 10461 USA

**Keywords:** Adjuvants, Cancer prevention

## Abstract

A critical aspect of cancer vaccine development is the formulation with effective adjuvants. This study evaluated whether combining a cationic plant-derived nanoparticle adjuvant (Nano-11) with the clinically tested STING agonist ADU-S100 (MIW815) could stimulate anticancer immunity by intradermal vaccination. Nano-11 combined with ADU-S100 (NanoST) synergistically activated antigen-presenting cells, facilitating protein antigen cross-presentation in vitro and in vivo. Intradermal vaccination using ovalbumin (OVA) as a tumor antigen and combined with Nano-11 or NanoST prevented the development of murine B16-OVA melanoma and E.G7-OVA lymphoma tumors. The antitumor immunity was abolished by CD8^+^ T cell depletion but not by CD4^+^ T cell depletion. Therapeutic vaccination with NanoST increased mouse survival by inhibiting B16-OVA tumor growth, and this effect was further enhanced by PD-1 checkpoint blockade. Our study provides a strong rationale for developing NanoST as an adjuvant for intradermal vaccination and next-generation preventative and therapeutic cancer vaccines by STING-targeted activation.

## Introduction

Tremendous progress in the research and development of therapeutic cancer vaccines has been made in the past decade. Cancer vaccines aim to achieve tumor regression, prevent disease recurrence, and generate long-lasting immunity against cancer while limiting off-target toxicity^1^. The activation of antigen-presenting cells (APCs), particularly dendritic cells (DCs), plays a crucial role in natural anticancer immunity. DCs capture and present tumor antigens to naive T cells, thereby initiating an effective tumor-specific T cell response^2,3^. Mature DCs migrate to secondary lymphoid organs, where they activate CD4^+^ helper T cells and CD8^+^ cytotoxic T lymphocytes (CTL) that specifically target tumors^2,3^.

Successful immunotherapy highly depends on the activation of CD8^+^ cytotoxic T lymphocytes (CTLs), which are critical components of the adaptive immune system’s anticancer responses^2,4,5^. Several immunotherapies have received FDA approval: immune checkpoint blockade that enhances T cell responses by releasing their inhibitory mechanisms^6,7^; tumor-specific CD8^+^ T cells with engineered chimeric antigen receptors (CARs)^8^; oncolytic viruses^9^; and the autologous therapeutic vaccine, sipuleucel-T (Provenge®), in which patient-derived DCs are loaded with prostate tumor antigen, stimulated with GM-CSF, and injected back into the patient to activate tumor-specific CD8^+^ T cells^10^. These immunotherapies have modernized cancer treatments, curing some patients and providing long-term remission for others. However, immunotherapies can be associated with adverse immune reactions and are inaccessible to many patients due to their high cost, emphasizing the need to optimize their efficacy and affordability. This is especially crucial when it comes to cancer vaccines since there is only one FDA-approved vaccine compared to nine immune checkpoint inhibitors and six CAR T cell therapies. Soluble protein antigens are poor at inducing cytotoxic T cell responses because uptake of proteins by pinocytosis is inefficient in generating peptides presented by MHC I^11^. Adjuvants can increase the efficiency of cross-presentation and inducing effective cytotoxic T cells by creating particulate antigens that are taken up by phagocytosis and by activating antigen-presenting cells^11,12^. Notably, the synergy of combination adjuvant systems stimulating diverse pathways could pave the way for enhanced vaccine-mediated immunity^13^.

Nanoparticle-based adjuvant systems offer an innovative approach for the advancement of next-generation preventive and therapeutic cancer vaccines and immunotherapy^14,15^. The customizable physical properties of nanoparticles present a versatile adjuvant platform that can facilitate the design of combination adjuvant systems for specific vaccine applications. This provides a dual adjuvant function: enhance vaccine immunogenicity while serving as a biocarrier for antigens and immunostimulants^16^. As a result, nanoparticle adjuvants can be utilized for the simultaneous delivery of antigens and immunopotentiators molecules to the same APC. Delivering the vaccine directly to APCs can reduce off-target toxicity by limiting the systemic diffusion of antigens and immunostimulatory compounds. While nanoparticle-based adjuvants show promise in vaccinology and immunotherapy, their application is challenged by the cost and complexities in the manufacturing process^17^.

The multifaceted platform of nanoparticles provides opportunities for the research and development of combination adjuvant systems, particularly in delivering small-molecule immunostimulants like stimulator of interferon genes (STING) agonists. Cyclic dinucleotides (CDNs) are STING agonists, which make them attractive candidates for cancer vaccine adjuvants as they induce a strong type I interferon (IFN) response^18^. Type I IFN plays a vital role in the immune response to cancer^19^ and promotes the cross-presentation of antigens for the activation of tumor-specific CD8^+^ T cells^20,21^. However, the use of CDNs as vaccine adjuvants is limited by the rapid diffusion of these small molecules from the injection site, which diminishes local activity and causes systemic (“wasted”) inflammation. Encapsulation of CDNs in nanoparticles can deliver the CDNs to DCs in the draining lymph nodes and limit their systemic distribution^22–25^. Most of these nanoparticles have limited immunostimulatory activity and function primarily as biocarriers. We recently developed a novel nanoparticle adjuvant, Nano-11, that is effective at stimulating antigen-specific humoral and cell-mediated immunity in mice and pigs and can also be used in combination with other immunostimulatory compounds such as the TLR3 agonist poly(I:C) and CDN^26,27^. Nano-11 is prepared from phytoglycogen, a widely available and inexpensive source, via two simple and easy-to-scale chemical processes resulting in nanoparticles with a diameter of approximately 70–80 nm and a positive surface charge^28^. Nano-11 stimulates DCs through the activation of NF-ĸB, the mitogen-activated protein kinase ERK, and the NLRP3 inflammasome^26,27^. Recent studies showed that the combination of cyclic-di-AMP (cdAMP) and Nano-11 has marked synergistic effects in the activation of DCs and the immune response to co-administered antigen^26^. In particular, the Nano-11/cdAMP combination adjuvant induced an increase in the number of antigen-specific IFN-γ^+^ and IL-17A^+^ CD4^+^ T cells (Th1 and Th17) and IFN-γ^+^ CD8^+^ T cells following intradermal vaccination with ovalbumin (OVA). While the activation of IFN-γ^+^ CD8^+^ T cells is suggestive of the cross-presentation of OVA, it remains to be determined whether these cells differentiate into effective and functional cytotoxic T cells, as this requires additional signals such as IL-12, type I IFN, and IL-6^29–31^. The current experiments were designed to determine the cytotoxicity and antitumor effect of the activated CD8^+^ T cells. In these experiments, cdAMP was replaced by ADU-S100 (MIW815), a synthetic analog with mixed phosphodiester linkages and a phosphorothioate backbone that provides protection from in vivo degradation and superior affinity to all known human STING alleles^32^. Clinical trials have demonstrated the safety of ADU-S100 in human patients^33^.

This is the first study to report the induction of functional anticancer immunity against murine melanoma and lymphoma tumors via intradermal vaccination utilizing a STING-based prophylactic and therapeutic cancer vaccine. We have demonstrated that NanoST, a novel adjuvant comprised of Nano-11 and ADU-S100, can be delivered intradermally to promote tumor-specific immunity and systemic protection. The findings from this investigation support the research and development of adjuvants in both vaccinology and immunotherapy, providing novel insights on the immune response to intradermal vaccination.

## Results

### Synergistic effects of Nano-11 and ADU-S100 on costimulatory molecules and cell signaling

**Fig. 1:**
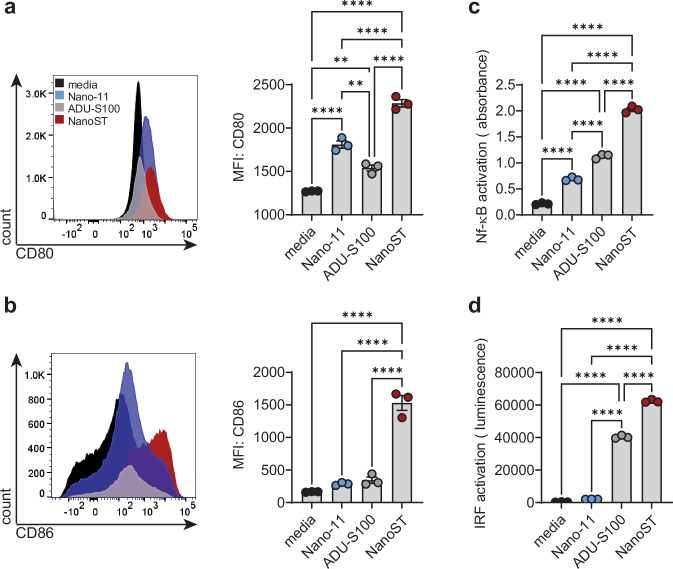
NanoST combination adjuvant synergy leads to enhanced CD80 and CD86 expression, NF-ĸB and IRF3 signaling pathway activation in human THP-1 cells. Flow cytometry analysis and median fluorescence intensity (MFI) of (**a**) CD80 and (**b**) CD86 expression on ADU-S100, Nano-11, and NanoST stimulated human THP-1 cells. **c** NF-κB activation and (**d**) IRF signaling in human THP1-Dual^TM^ cells stimulated with ADU-S100, Nano-11, and NanoST. The bars represent the mean ± SEM of three replicates. The data depicted is representative from one of two independent experiments. The statistical significance of differences between experimental groups was determined by one-way ANOVA with Tukey’s multiple comparisons test. **p < 0.01, ****p < 0.0001.

To determine if Nano-11 synergizes with ADU-S100, we first tested the effect on the expression of the costimulatory molecules CD80 and CD86 by human THP-1 cells. Nano-11 and ADU-S100 alone induced CD80 expression in human THP-1 cells, while stimulation with the NanoST combination adjuvant further enhanced CD80 expression (Fig. 1a). NanoST stimulation resulted in significantly increased CD86 expression in THP-1 cells, while neither Nano-11 nor ADU-S100 alone had any effect (Fig. 1b). To further examine the signaling pathways activated by Nano-11, ADU-S100, and NanoST, we used the NF-κB-SEAP and IRF-Lucia luciferase THP1-Dual reporter cell line (Fig. 1c, d). Stimulation with ADU-S100 alone activated NF-κB and IRF3 in THP1-Dual cells. While Nano-11 promoted NF-κB activation but not IRF3 activation, the NanoST combination adjuvant produced a significant synergistic effect and elicited substantially higher activation of both signaling pathways. Together, these in vitro studies show that the combination of Nano-11 and ADU-S100 had marked synergistic effects on the activation of antigen-presenting cells.

### Intradermal vaccination with NanoST induces an effective systemic cell-mediated and humoral immune response

**Fig. 2:**
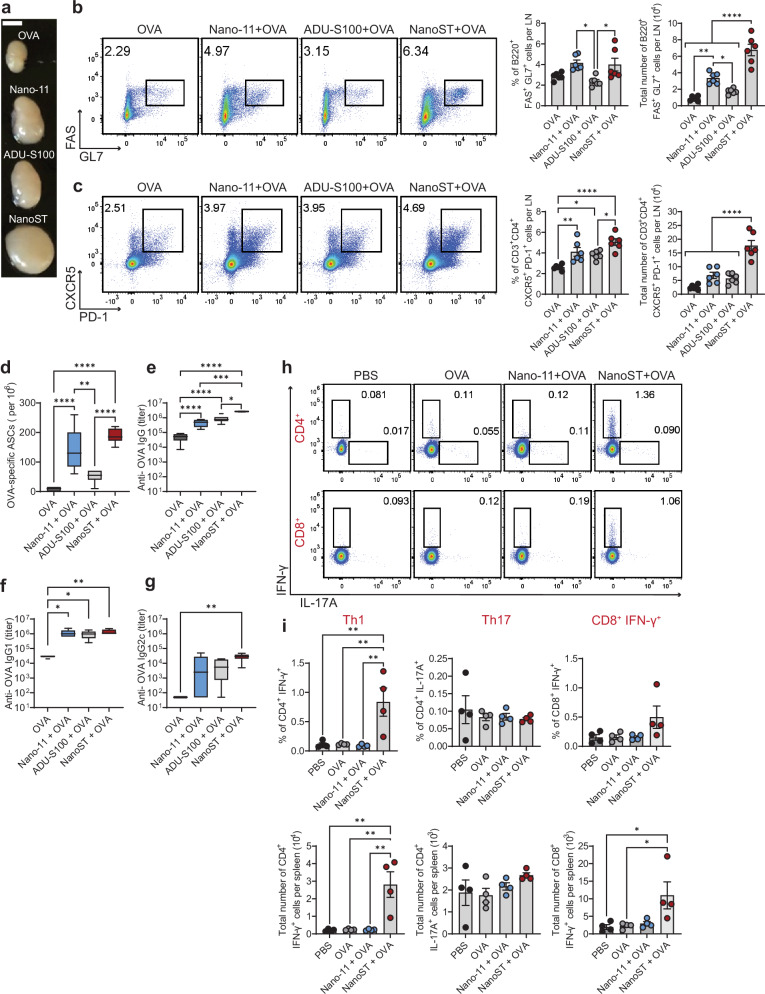
Intradermal vaccination with NanoST induces germinal center (GC) responses and the development of effector T cells. **a** Superficial parotid lymph nodes 10 days after intradermal vaccination with OVA, Nano-11 + OVA, ADU-S100 + OVA, or NanoST + OVA. Scale bar, 2 mm. Bivariate density plots displaying the frequencies and absolute numbers of (**b**) GC B cells (B220^+^ FAS^+^ GL7^+^) and (**c**) GC Tfh cells (CD3^+^ CD4^+^ CXCR5^+^ PD-1^+^) 10 days following the second injection with the specified vaccines. **d** OVA-specific antibody secreting cells (ASCs) and serum anti-OVA (**e**) IgG, (**f**) IgG1, and (**g**) IgG2c titers after intradermal vaccination. Four to six mice were used to generate the data for each group, and the box and whisker plots show the median 25th–75th percentiles. **h** Bivariate density graphs demonstrating the CD4^+^ and CD8^+^ T cell frequencies of isolated splenocytes from mice that were OVA-restimulated for 24 h. **i** Absolute numbers of IFN-γ^+^ and IL-17A^+^ secreting CD4^+^ and IFN-γ^+^ CD8^+^ cells were quantified. The bars represent the mean ± SEM of four mice per group. The data depicted is representative of two independent experiments. The statistical significance of differences between experimental groups was determined by one-way ANOVA with Tukey’s multiple comparisons test. *p < 0.05, **p < 0.01, ***p < 0.001, ****p < 0.0001.

To investigate the immunogenicity of the NanoST adjuvant system, mice were intradermally immunized with OVA alone, OVA adsorbed to Nano-11, ADU-S100, or NanoST (Supplementary Fig. 1). No injection site reactions were noted with any of the intradermal vaccinations consistent with our previous observations that utilized the Nano-11 and NanoST adjuvants in mice and swine^26,34^. Mice vaccinated with Nano-11, ADU-S100, or NanoST had enlarged draining lymph nodes (dLNs; superficial parotid) 10 days after the second intradermal injection of the ear pinna (Fig. 2a). Flow cytometric analysis was performed on the dLNs to quantify the number of germinal center B (GCB) cells **(**Fig. 2b) and follicular helper T (Tfh) cells (Fig. 2c). Nano-11 and NanoST induced a significant increase in the proportion and number of GCB cells; however, only the NanoST combination adjuvant showed an increase in both the percentage and number of Tfh cells. Nano-11 alone induced the differentiation of antigen-specific antibody-secreting cells (ASCs; plasma cells) in the bone marrow (Fig. 2d), which was significantly enhanced when Nano-11 and ADU-S100 were combined. Both Nano-11 and ADU-S100 alone enhanced anti-OVA total IgG and IgG1 titers (Fig. 2e, f); however, the NanoST combination adjuvant significantly increased both titers in addition to inducing the production of IgG2c (Fig. 2g).

The number of antigen-specific effector CD4^+^ and CD8^+^ T cells was evaluated by intracellular cytokine detection following re-stimulation of splenocytes with OVA. Only mice vaccinated with NanoST had a significant increase in the frequency and total number of OVA-specific IFN-γ-secreting CD4^+^ and CD8^+^ T cells (Fig. 2h, i), whereas the number of IL-17A^+^ T cells was not affected. Thus, intradermal immunization with the NanoST combination adjuvant elicited a robust antigen-specific humoral and cell-mediated immune response. The activation of OVA-specific CD8^+^ IFN-γ^+^ T cells after intradermal vaccination with NanoST indicates cross-presentation of OVA-derived peptides.

### NanoST stimulates the differentiation of antigen-specific CD8^+^ T cells

**Fig. 3:**
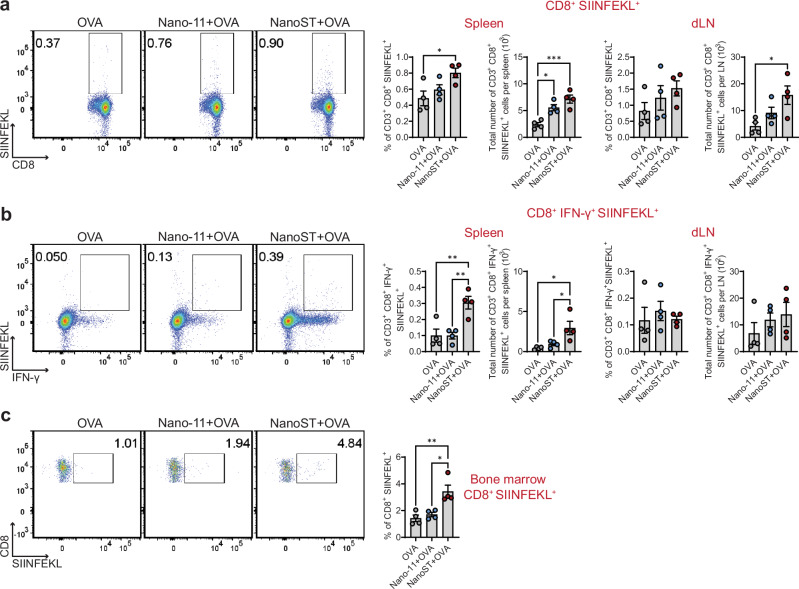
NanoST administered intradermally stimulates the expansion of SIINFEKL-specific effector CD8^+^ T cells. The intradermal vaccination of OVA, Nano-11 + OVA, or NanoST + OVA was conducted on C57BL/6 J mice on day 1 and day 21. The superficial parotid draining lymph node (dLN) located near the intradermal injection site, spleen, and bone marrow were collected on day 31 to quantify the CD3^+^ CD8^+^ effector T cell response with SIINFEKL-specificity. Bivariate density plots displaying the frequencies and absolute numbers of (**a**) SIINFEKL^+^ CD8^+^ T cells, and (**b**) SIINFEKL^+^ IFN-γ^+^ CD8^+^ T cells. **c** Bone marrow cells were isolated from the left femur and tibia of each mouse and screened for SIINFEKL^+^ CD8^+^ T cells. The H-2K^b^ chicken OVA_257–264_ SIINFEKL tetramer was used to identify SIINFEKL-specific CD8^+^ T cells. The statistical significance of differences between groups was determined by one-way ANOVA with Tukey’s multiple comparisons test. *p < 0.05, **p < 0.01, ***p < 0.001.

Our next aim was to determine the distribution of OVA-specific CD8^+^ T cells by utilizing a H-2K^b^ SIINFEKL-specific MHC class I tetramer. Mice were immunized with OVA, Nano-11 + OVA, or NanoST + OVA and 10 days post-secondary vaccination the draining lymph nodes (dLNs; superficial parotid), spleen and bone marrow were collected. There was a modest increase in the number of SIINFEKL^+^ CD8^+^ T cells in the spleen of Nano-11 + OVA immunized mice and a greater increase in both the spleen and dLN following vaccination with the NanoST adjuvant (Fig. 3a). Only mice vaccinated with NanoST had an expansion of effector SIINFEKL^+^ IFN-γ-secreting CD8^+^ T cells in the spleen (Fig. 3b). Furthermore, the frequency of SIINFEKL^+^ CD8^+^ T cells was increased in the bone marrow of NanoST + OVA vaccinated mice, indicating their differentiation into effector-memory CD8^+^ T cells (Fig. 3c). The increased number of SIINFEKL^+^ IFN-γ^+^ effector CD8^+^ T cells suggest that the NanoST + OVA vaccination enhances the cross-presentation of OVA and differentiation of antigen-specific CD8^+^ T cells.

### DCs primed with Nano-11 or NanoST cross-present and activate antigen-specific CD8^+^ T cells in vitro

**Fig. 4:**
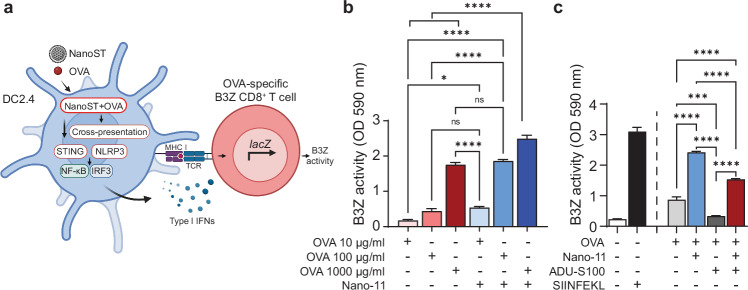
Nano-11 and NanoST enhance cross-presentation by dendritic cells. **a** DC2.4 and B3Z cross-presentation in vitro model. **b** DC2.4 cells were incubated with 10, 100, or 1000 μg/ml of soluble OVA or OVA adsorbed to Nano-11 and co-cultured with OVA-specific B3Z CD8^+^ T cells. The activation of B3Z cells was determined as β–galactosidase activity at 590 nm (OD 590). **c** Stimulation of DC2.4 cells with 10 μg/ml OVA alone or with Nano-11 or the NanoST combination adjuvant. SIINFEKL (0.5 μg/ml) and media served as the positive and negative controls, respectively. The bars represent the mean ± SEM of four replicates. The data shown are representative of two independent experiments. Created with BioRender.com. The statistical significance of differences between experimental groups was determined by one-way ANOVA with Tukey’s multiple comparisons test. ns, not statistically significant. *p < 0.05, ***p < 0.001, ****p < 0.0001.

The ability of Nano-11 and NanoST to facilitate cross-presentation of OVA was assessed in vitro utilizing DC2.4 dendritic cells and B3Z hybridoma T cells (Fig. 4a). B3Z hybridoma cells recognize the OVA_257–264_ (SIINFEKL) peptide presented by H2-K^b^, resulting in activation and lacZ expression independent of costimulatory molecules, and can be used as a read-out for cross-presentation^35^. The DC2.4 cells were first incubated with increasing concentrations of OVA with or without Nano-11, followed by co-culture with the OVA_257–264_ (SIINFEKL)-specific B3Z *lacZ* inducible CD8^+^ T cells (Fig. 4b). Although OVA alone could induce cross-presentation, especially at a high concentration of 1 mg/ml, the cross-presentation was significantly enhanced when OVA was adsorbed to Nano-11. In contrast, ADU-S100 decreased the cross-presentation of soluble OVA and reduced the stimulatory effect of Nano-11 (Fig. 4c). Nonetheless, the cross-presentation of OVA was enhanced when delivered with NanoST compared to OVA alone, indicating that both Nano-11 and NanoST supported cross-presentation of OVA via a direct effect on dendritic cells.

### NanoST-adjuvanted intradermal immunizations induces antigen-specific lysis of target cells

**Fig. 5:**
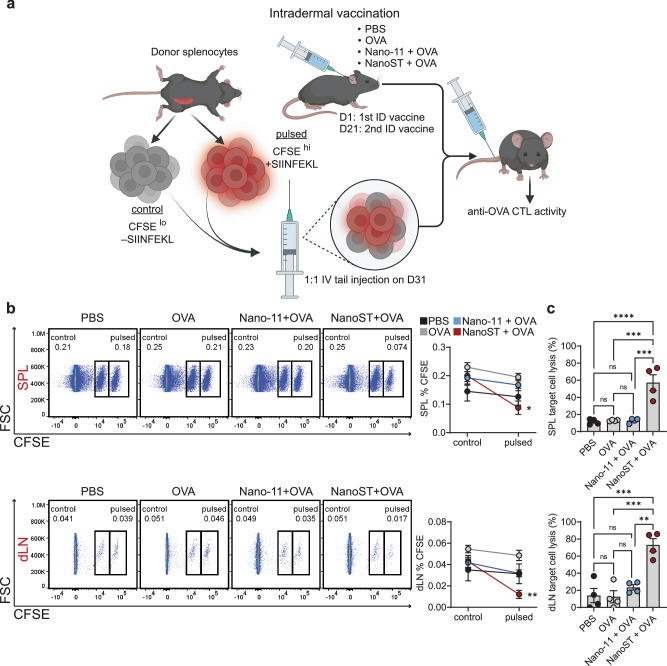
Intradermal vaccination with NanoST induces antigen-specific cytotoxic T cells that mediate the lysis of SIINFEKL-labeled target cells. **a** Mice were primed and boosted with PBS, OVA, Nano-11 + OVA, or NanoST + OVA. At 10 days after the second immunization, all the mice were intravenously injected with donor splenocytes that had been fluorescently labeled 1:1 with CFSE^hi^ + SIINFEKL (pulsed) or CFSE^low^ – SIINFEKL (control). **b** Lysis of CFSE^hi^ donor splenocytes pulsed with SIINFEKL was assessed by flow cytometry in the spleen (SPL) and from the superficial parotid draining lymph node (dLN) located near the intradermal injection site. **c** The percentage of lysed target cells in each vaccinated mouse was determined by calculating the difference between the pulsed and control populations in the vaccinated mice and comparing them to the PBS group. The bars represent the mean ± SEM of four replicates. The flow cytometry data shown is representative of one animal from each experimental group. Created with BioRender.com. The statistical significance of differences between experimental groups was determined by a two-way ANOVA with Sidak’s multiple comparison test (**b**) or a one-way ANOVA with Tukey’s multiple comparison test (**b**). *p < 0.05, **p < 0.01, ***p < 0.001, ****p < 0.0001.

The functional activity of the OVA-specific CD8^+^ T cells induced by intradermal vaccination with Nano-11 or NanoST was determined by assessing their ability to kill SIINFEKL-pulsed target cells (Fig. 5a). Donor splenocytes were divided into two groups, CFSE^hi^-labeled splenocytes pulsed with SIINFEKL and non-pulsed CFSE^low^-labeled splenocytes, and intravenously injected into the vaccinated mice. Bivariate density plots of isolated cells from the spleen and dLN of mice immunized with NanoST + OVA showed a significant reduction in the percentage of SIINFEKL-pulsed donor cells (Fig. 5b). Quantitative analysis of the proportion of lysed target cells in mice immunized with the NanoST combination adjuvant indicated that NanoST, but not Nano-11, induced a significant increase of antigen-specific CTLs (Fig. 5c).

### Intradermal vaccination with Nano-11 and NanoST prevents the growth of tumors

**Fig. 6:**
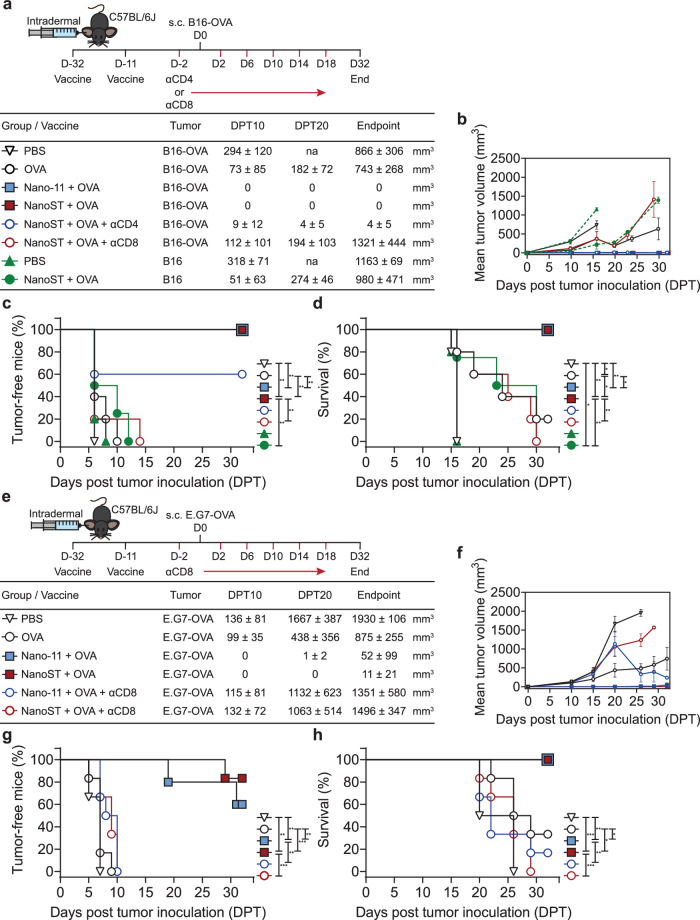
The Nano-11 and NanoST antitumor-mediated prophylactic protection is dependent on CD8^+^ T cells in both melanoma and lymphoma cancer models. **a** Melanoma vaccination strategy, T cell depletion schedule, and tumor volume mean ± SEM (*n* ≥ 5) at indicated days post tumor (DPT) inoculation. The right flank of C57BL/6 J mice were inoculated with 1 × 10^6^ B16-OVA or B16 tumor cells by subcutaneous (s. c.) injection; na, not available, which indicates no animals reached DPT20. **b** Mean tumor growth curves (mm^3^) in each group. **c** Percentage of B16-OVA or B16 tumor-free mice and (**d**) survival rate post tumor inoculation. (**e**) Lymphoma vaccination strategy, T cell depletion schedule, and tumor volume mean ± SEM (*n* ≥ 5) at different days post tumor (DPT) inoculation with 2.5 × 10^6^ E.G7-OVA tumor cells. **f** Mean tumor growth curves (mm^3^). **g** Percentage of E.G7-OVA tumor-free mice and (**h**) survival rate. The log-rank (Mantel-Cox) curve comparison test was applied to determine the percentage of tumor-free mice and their survival. *p < 0.05, **p < 0.01, ***p < 0.001.

The previous experiments showed that intradermal vaccination with Nano-11 or NanoST can elicit an effective antigen-specific humoral and cell mediated response with functional cytotoxic T cells. These results led us to evaluate Nano-11 and NanoST as adjuvant platforms for STING-targeted cancer immunotherapy in mouse models of melanoma and lymphoma, in which OVA functions as a tumor-specific antigen. Mice were immunized twice intradermally with a three-week interval, followed by subcutaneous inoculation with B16, B16-OVA (Fig. 6a), or E.G7-OVA (Fig. 6e) tumor cells. By day 10, all PBS- and OVA-vaccinated control mice had developed tumors. Mice immunized with Nano-11 + OVA or NanoST + OVA were completely protected against the development of tumors when inoculated with B16-OVA melanoma cells. In contrast, immunization with NanoST + OVA delayed but did not prevent the progression of tumor growth in B16 melanoma cells that lack OVA (Fig. 6b, c). Although a few mice immunized with Nano-11 + OVA or NanoST + OVA developed E.G7-OVA lymphomas, the tumors in these mice were small, and the majority of mice remained tumor-free (Fig. 6f, g). All mice immunized with Nano-11 and NanoST survived the 32-day observation period after inoculation with B16-OVA or E.G7-OVA (Fig. 6d, h).

Depletion of CD8^+^ T cells after the two-dose immunization abolished the protection against B16-OVA and E.G7-OVA elicited by Nano-11 or NanoST. In contrast, depletion of CD4^+^ T cells did not significantly affect the efficacy of immunization with NanoST + OVA. These observations indicate that the antitumor protection induced by intradermal vaccination with the Nano-11 and NanoST adjuvants was dependent on the activation and expansion of tumor-specific CTLs. Overall, we demonstrated the immunoprophylactic potential of cancer vaccines formulated with Nano-11 and NanoST in preventing the formation of melanoma and lymphoma tumors. Following this, the next objective was to evaluate the therapeutic efficacy of Nano-11 and NanoST in treating established tumors.

### Therapeutic immunity from intradermal vaccination with Nano-11 and NanoST

**Fig. 7:**
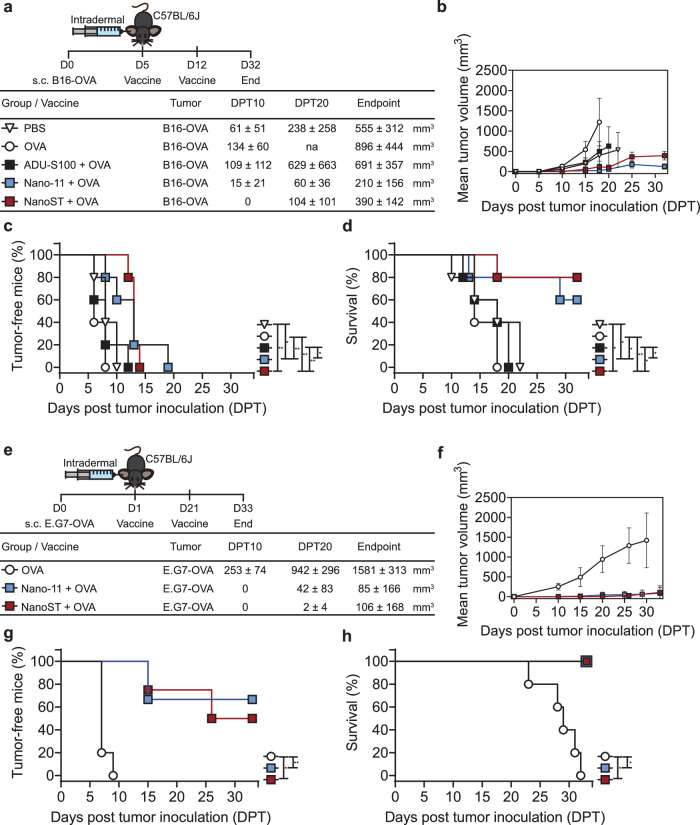
Immunotherapeutic capacity of intradermal vaccination with Nano-11 or NanoST enhanced survival rates and suppressed tumor progression. **a** Vaccination schedule for the treatment of B16-OVA melanoma and tumor volume mean ± SEM (n ≥ 5) at indicated days post tumor (DPT) inoculation with 1.75 × 10^5^ B16-OVA cancer cells by s. c. injection; na, not available, no animals reached DPT20. **b** Mean tumor growth curves over 32 days (mm^3^). **c** Percentage of tumor-free mice and (**d**) survival rate of mice. **e** Lymphoma therapeutic cancer vaccine schedule and tumor volume mean ± SEM (n ≥ 5) at indicated days post tumor (DPT) inoculation. C57BL/6 J mice were inoculated in the right flank with 2.5 × 10^6^ E.G7- OVA. **f** Mean tumor growth curves (mm^3^). **g** Percentage of tumor-free mice and (**h**) long-term survival of mice. The log-rank (Mantel-Cox) test was used to evaluate the proportion of tumor-free mice and the survival rate. *p < 0.05, **p < 0.01.

The immunotherapeutic efficacy of the Nano-11 and NanoST cancer vaccines was assessed in melanoma and lymphoma tumor models. The therapeutic regimen with B16-OVA cells was performed by intradermal vaccinations at 5 and 12 days after tumor inoculation (Fig. 7a). Vaccination with Nano-11 + OVA and NanoST + OVA significantly suppressed tumor development, in contrast to injection of PBS (control), OVA, or ADU-S100 + OVA alone (Fig. 7b, c). Furthermore, mice injected with PBS, OVA, or ADU-S100 + OVA had significantly lower survival rates than Nano-11 and NanoST vaccinated mice (Fig. 7d). The immunotherapeutic potential of the Nano-11 and NanoST adjuvants was also assessed in the E.G7-OVA lymphoma cancer model. Mice were vaccinated intradermally with OVA, Nano-11 + OVA, or NanoST + OVA at 1 and 21 days after subcutaneous inoculation with E.G7-OVA cells (Fig. 7e). Intradermal immunization with Nano-11 and NanoST resulted in inhibition of E.G7-OVA growth (Fig. 7f). Notably, at day 30, 70% and 50% of mice vaccinated with Nano-11 and NanoST, respectively, were free of E.G7-OVA tumors, compared with 100% tumor formation in OVA-vaccinated mice by day 10 (Fig. 7g). The intradermal administration of Nano-11 or NanoST vaccines resulted in a 100% survival over the 32 days observation period (Fig. 7h).

### Therapeutic immunity by intradermal vaccination with NanoST combined with PD-1 blockade

**Fig. 8:**
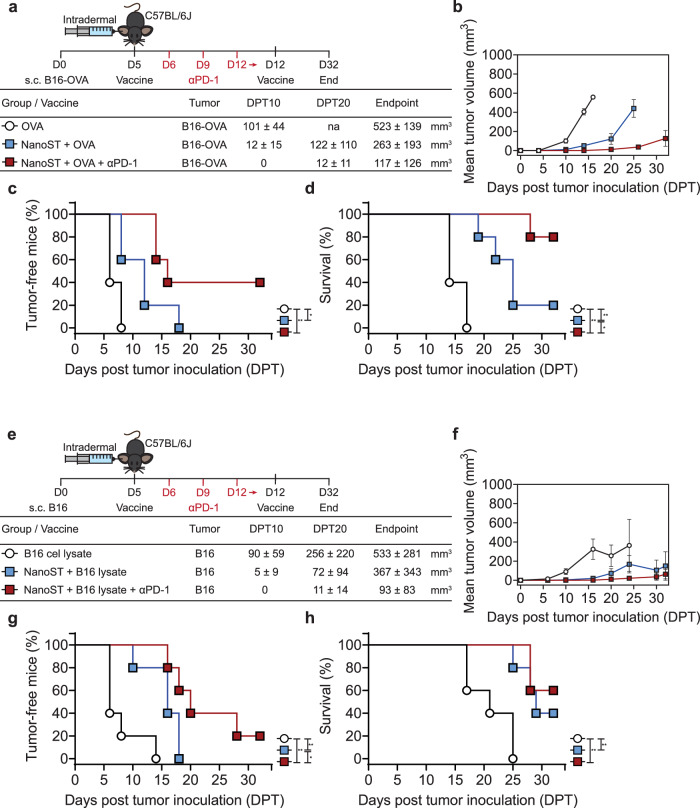
Combination immunotherapy with NanoST-based cancer vaccines and PD-1 checkpoint blockade. **a** Antigen-specific vaccination strategy, αPD-1 immunotherapy schedule, and tumor volume mean ± SEM (*n* ≥ 5) at indicated days post tumor (DPT) inoculation. C57BL/6 J mice were inoculated in the right flank by subcutaneous (s. c.) injection with 3.5 × 10^5^ B16-OVA cancer cells; na, not available, no animals reached DPT20. **b** Mean tumor growth curves (mm^3^). **c** Percentage of tumor-free mice and (**d**) survival rate. **e** Whole cell lysate cancer vaccine schedule, αPD-1 immunotherapy regimen, and tumor volume mean ± SEM (*n* ≥ 5) at indicated days post tumor (DPT) inoculation. Mice were subcutaneously inoculated with 3.5 × 10^5^ B16 cancer cells. **f** Mean tumor growth curves (mm^3^), (**g**) percentage of mice without palpable B16 tumors, and (**h**) survival rate of mice. The log-rank (Mantel-Cox) test was used to evaluate the proportion of tumor-free mice and the survival rate. *p < 0.05, **p < 0.01.

We utilized a PD-1 checkpoint inhibitor in an effort to maximize the therapeutic efficacy of NanoST. Mice were subcutaneously inoculated with B16-OVA cells, followed by two intradermal vaccinations with NanoST + OVA on day 5 and 12 (Fig. 8a). The treatment of anti-PD-1 was scheduled on day 6, 9, and 12. Combination immunotherapy with PD-1 blockade and NanoST vaccination inhibited the development of the B16-OVA tumors (Fig. 8b, c). These results show that the immunotherapeutic protection induced by intradermal vaccination with OVA and NanoST is further enhanced by the co-administration of the PD-1 checkpoint inhibitor (Fig. 8d).

As the identity of tumor antigens in human cancer patients is often unknown, we investigated the immunotherapeutic potential of NanoST-adjuvanted whole cell lysate vaccines. Mice bearing B16 tumors were intradermally vaccinated with NanoST + B16 cell lysate on days 5 and 12 (Fig. 8e). Anti-PD-1 was administered on days 6, 9, and 12. (Fig. 8f–h). Addition of the PD-1 checkpoint blockade synergized with the NanoST + B16 cell lysate vaccine to increase the tumor-free survival rate of mice (Fig. 8g).

This data provides evidence for the efficacy and versatility of the NanoST-adjuvant system to intradermally deliver tumor-associated antigens to induce tumor-specific humoral and cell-mediated immunity with both prophylactic and therapeutic protection. Together, this is the first investigation to show the potential of intradermal vaccination as an innovative avenue for the research and development of next-generation vaccines against both infectious diseases and cancer.

## Discussion

The stimulator of IFN genes (STING) pathway is an essential component in the activation of antigen-presenting cells (APCs), resulting in enhanced production of type I IFN and proinflammatory cytokines that induce the differentiation of tumor-specific CD8^+^ T lymphocytes^19^. The activation of STING as a cancer therapeutic has paved the way for the development of numerous pharmacologic-class STING agonists that are now in clinical testing^36^. ADU-S100 (MIW815) is a first generation synthetic cyclic dinucleotide (CDN) STING agonist that was designed to be more chemically stable and to have a higher affinity for all human STING alleles^32^. Intratumoral injection of ADU-S100 leads to the generation of tumor-specific CD8^+^ T cells in different mouse cancer models^37^. Recent clinical studies that evaluated the efficacy and safety of intratumoral immunotherapy with ADU-S100 found that there were no dose-limiting toxicities in patients with metastatic solid tumors or lymphomas. The outcomes of these trials confirmed that ADU-S100 is safe and well tolerated in patients; however, the limited anticancer efficacy suggests suboptimal STING activation by ADU-S100. This could be caused by in vivo degradation, rapid diffusion from the injection site, and induction of apoptosis in T cells^22,38^. The delivery of cyclic dinucleotides (CDNs) to myeloid cells can be improved by encapsulation in liposomes; however, the encapsulation efficiency is low^22,24^. We previously reported that negatively charged CDNs efficiently adsorb to the cationic Nano-11 nanoparticles and that intradermal vaccination with Nano-11-based vaccines increased antigen uptake and transport by migratory DCs into draining lymph nodes^26^. Here we provide evidence of effectively delivering ADU-S100 in vitro and in vivo by adsorbing it to Nano-11. The combination of Nano-11 with ADU-S100 (NanoST) synergizes to stimulate complementary signaling pathways in DCs, resulting in enhanced adjuvanticity effects as shown by the increased expression of the costimulatory molecules CD80 and CD86 and the activation of NF-ĸB and IRF3. The NanoST combination adjuvant stimulated the differentiation and function of Tfh cells and GC B cells, which are critical for the generation of high-affinity antibodies that can contribute to cancer control^39,40^. Indeed, expansion of the number of Tfh and GC B cells is associated with long-term survival in patients with colorectal or breast cancer^41,42^.

Tumor eradication correlates with the polarization and proliferation of Th1 cells and the activation of CD8^+^ cytotoxic T lymphocytes (CTLs), whereas the role of Th17 cells is controversial and dependent on the tumor type and environment^43,44^. The NanoST adjuvant significantly enhanced the differentiation of IFN-γ secreting Th1 cells and CTLs but did not induce a Th17 response. We previously reported that Nano-11 combined with the natural STING agonist cyclic-di-AMP induced Th17 cells in addition to a strong Th1 response^26^, but these experiments were conducted with BALB/c mice which have a stronger tendency towards Th17 responses compared to C57BL/6 mice^45^. The increased IgG2c antibody titers in mice immunized with OVA and NanoST further supports the induction of Th1 responses. In addition, NanoST stimulated IgG1 production which aligns with CD4^+^ Th2 cells indicating an overall mixed Th1/Th2 profile. Following immunization with NanoST and OVA, SIINFEKL^+^ CD8^+^ T cells were detected in the spleen, lymph nodes, and bone marrow. The population of OVA-specific CD8^+^ T cells in the bone marrow likely includes both circulating CD8^+^ T cells and resident memory cells. The bone marrow has previously been identified as a site for the accumulation of memory CD8^+^ T cells following systemic immunization^46,47^ where they can remain as resting cells for over a year^48^.

A high concentration of soluble OVA induced cross-presentation, which can be attributed to the mannosylation of OVA, as this enables uptake via the mannose receptor^49^. Nano-11 efficiently enhanced the cross-presentation of OVA, whereas ADU-S100 inhibited the cross-presentation of both soluble OVA and Nano-11 adsorbed OVA in vitro. This is likely caused by activation of the DCs, which downregulates the uptake of antigens, as demonstrated for activation of DCs via Toll-like receptors^50,51^. In contrast with these in vitro results, the in vivo experiments revealed that NanoST induced a significant expansion of IFN-γ-secreting CD8^+^ T cells and cytotoxic T cell activity, which was not observed for Nano-11. Nevertheless, intradermal immunization of mice with either Nano-11 or NanoST prevented the development of tumors, which were both dependent on CD8^+^ T cells. This suggests that Nano-11, like NanoST, could induce the cross-presentation and activation of cytotoxic T cells, but the number of T cells activated by Nano-11 was lower and below the limit of detection using the flow cytometric assays. This suggests that, while Nano-11 induced cytotoxic T cell activity, NanoST is superior in the induction of CD8^+^ cytotoxic T cells, which is likely caused by the activation of DCs by multiple complementary activation pathways. The immunotherapeutic protection induced by immunization with OVA and NanoST was significantly improved with the administration of an anti-PD-1 checkpoint inhibitor. This suggests increased expression of PD-L1 by the B16 melanoma cells which blocks the activation of CD8^+^ tumor-infiltrating T cells^52^. The beneficial effect of anti-PD-1 antibodies reflects the robust activation of IFN-γ-secreting CD4^+^ and CD8^+^ T cells by NanoST because IFN-γ induces the expression of PD-L1 on tumor cells^53^. Blockade of the PD-1/PD-L1 interaction releases the inhibition of the tumor-infiltrating, vaccine-induced CD8^+^ T cells.

The majority of our experiments used OVA and OVA-transfected tumor cells in which OVA serves as a tumor-specific antigen. However, tumor-specific antigens vary between individual patients and their identification is laborious and costly in spite of tremendous advances in high throughput sequencing technologies. A simpler method is to prepare tumor vaccines from whole tumor cells which does not require identification of specific antigens and has the advantage of providing multiple antigens to stimulate a robust CD4^+^ and CD8^+^ T cell response^54^. Repeated freeze-thaw cycles induces cell necrosis which releases immunogenic molecules^54,55^. While injection of the necrotic tumor cell lysate alone induced a modest increase in survival, combination with NanoST significantly enhanced the survival of the mice. This suggests that the NanoST combination adjuvant has practical applications for preventive and therapeutic cancer vaccines.

In summary, the Nano-11 nanoparticle adjuvant and the STING agonist ADU-S100 have complementary and synergistic immunostimulatory effects, resulting in robust activation of DCs by the NanoST combination adjuvant. Intradermal delivery of vaccines formulated with NanoST induces the proliferation of antigen-specific GC B cells and the differentiation of Tfh and Th1 CD4^+^ T cells. The vaccine induced effective cross-presentation of protein antigen, generating an antigen-specific CTL response that suppressed the development of tumors in a preventive and therapeutic manner. These studies support the further investigation of NanoST as an innovative adjuvant system for the development and delivery of cancer vaccines.

## Methods

### Ethics statement

The Purdue University Animal Care and Use Committee approved all experiments. All experiments performed on animals were in accordance with current guidelines and regulations. Six- to eight-week-old female C57BL/6 J (RRID: IMSR_JAX:000664) mice were purchased from the Jackson Laboratory (Bar Harbor, ME). They were housed in ventilated cages with ad libitum access to food and water, with three to four animals per box. The room was kept at 20 ± 2 °C and 50 ± 15% relative humidity, with a light/dark cycle of 12 h. An acclimatization period of one week was given to the mice before the onset of the experiments. Mice received 20 μl of vaccine through intradermal injection into the pinna of the ear. The vaccines were administered again after 7 or 21 days. Inhalational isoflurane was used to induce anesthesia in mice. The Purdue University Animal Care and Use Committee approved the euthanasia of all animals using the carbon dioxide (CO_2_) inhalation method, followed by cervical dislocation. Blood and tissue samples were collected 10 days after the second immunization, unless otherwise specified.

### Vaccine formulations

Nano-11 was prepared as previously described^28^. Briefly, in two sequential chemical processes, phytoglycogen (PG) nanoparticles from sweet corn encoding the *sugary-1* mutant gene were conjugated with octenyl succinic anhydride and (3-chloro-2-hydroxypropyl)-trimethylammonium chloride to create PG-OS-CHPTAC (Nano-11). Endotoxin-free OVA was purchased from InvivoGen (San Diego, CA) and ADU-S100 from ChemiTek (MIW815; Indianapolis, IN). Each reagent was resuspended in a sterile solution consisting of 10 mm Tris-saline (pH 7.4). The adsorption efficiency of ADU-S100 to Nano-11 is over 80%, as determined by ultraperformance liquid chromatography/tandem mass spectrometry^56^. The intradermal vaccines had a final volume of 20 μl and were formulated by first combining 4 mg/ml Nano-11 with 250 μg/ml ADU-S100 to make the combination adjuvant (NanoST) for 1 h at room temperature, followed by the adsorption of 500 μg/ml OVA to Nano-11, ADU-S100, or NanoST for 1 h at room temperature. The final adjuvant and antigen doses per vaccine consisted of Nano-11 (80 μg), ADU-S100 (5 μg), OVA (10 μg), or B16 lysate (50 μg).

A B16 (RRID: CVCL_0158) whole lysate was prepared by freeze-thaw cell lysis. B16 cells were collected, washed five times with sterile phosphate-buffered saline (PBS) buffer, and assessed for their viability. Cell lysis was achieved by transferring 1 ml of cells (9 × 10^6^ cells per ml) to a sterile cryogenic vial and subjecting it to eight freeze-thaw cycles between the gas phase of liquid nitrogen (4 min) and a 37 °C (5 min) water bath. The cells were centrifuged at 300 × *g* for 5 min, and the supernatant was filtered through a 40-micron cell strainer (Corning, Corning, NY). A micro-BCA protein assay kit (Thermo Fisher Scientific, Waltham, MA) was utilized to determine the protein concentration of the lysate. Aliquots of the lysate were stored at −80 °C. The B16 lysate vaccine was formulated by mixing 2.5 mg/ml of B16 lysate to NanoST for 1 h at room temperature.

The physical characterization of the vaccine formulations was determined by utilizing a zetasizer (Nano ZS90, Malvern, UK) as previously described^57^. Briefly, the Nano-11 ± ADU-S100 ± OVA or B16 lysate vaccines were prepared as described above. A 0.1% and 0.01% of the final vaccine concentration in 10 mM Tris-saline (pH 7.4) were used to measure the particle size and zeta potential, respectively. Each test group consisted of three independent vaccine formulations. Three readings were taken for each of the vaccine formulations to determine the Z-average hydrodynamic diameter and zeta-potential measurements.

### Cell lines

DC2.4 cells (RRID: CVCL_J409)^58^ were generously donated by Dr. Kenneth Rock (UMass Chan Medical School, MA). Dr. Marulasiddappa Suresh (University of Wisconsin-Madison, WI) kindly supplied the B3Z CD8^+^ T cell hybridoma (RRID: CVCL_6277)^59,60^. DC2.4 and B3Z cells were cultured in complete RPMI (RPMI 1640 supplemented with 2 mm L-glutamine, 55 μm 2-mercaptoethanol, 1× non-essential amino acids, 10 mm HEPES, 100 U/ml penicillin, 100 μg/ml streptomycin, and 0.25 μg/ml amphotericin) with 10% FBS. The B3Z cells were kept under selection by supplementing the culture media with 500 μg/ml geneticin (G418 sulfate, Thermo Fisher Scientific, MA). The OVA-expressing B16-OVA (RRID: CVCL_WM78; generously donated by Dr. Matthew Olson, Purdue University) and EG.7-OVA tumor cells (RRID: CVCL_3505) were maintained in a selection environment with 0.4 mg/ml of G418 in RPMI 1640 supplemented with 2 mm L-glutamine, 55 μm 2-mercaptoethanol, 10 mm HEPES, 100 U/ml penicillin, 100 μg/ml streptomycin, and 0.25 μg/ml amphotericin, 4.5 g/L glucose, 1 mm sodium pyruvate, and 10% FBS.

Human THP-1 cells (TIB-202; American Type Culture Collection, Manassas, VA) were cultured in complete RPMI with 10% FBS. Cells were seeded at a density of 5 × 10^5^ cells per well in a 96-well plate, stimulated with Nano-11 (80 μg/ml) and ADU-S100 (5 μg/ml) for 24 and 48 h at 37 °C with 5% CO_2_, and then processed for flow cytometry analysis. Similarly, the NF-ĸB-SEAP and IRF-Luc reporter THP1-Dual cells (RRID: CVCL_X599; InvivoGen, San Diego, CA) were grown at a density of 1 × 10^5^ cells per well in a 96-well plate in complete RPMI supplemented with 10% FBS, 100 μg/ml zeocin, 100 μg/ml normocin, and 10 μg/ml blasticidin. THP1-Dual cells were subsequently stimulated with Nano-11 (80 μg/ml) and ADU-S100 (5 μg/ml) for 24 and 48 h at 37 °C with 5% CO_2_, and the supernatants were collected to quantify activation of the NF-ĸB and IRF pathways.

### Antigen cross-presentation in vitro

The B3Z CD8^+^ T cell hybridoma cell line that expresses β-galactosidase under the control of the IL-2 promoter was utilized to detect antigen cross-presentation of the OVA _257–264_ (SIINFEKL, InvivoGen, San Diego, CA) peptide^59^. Briefly, DC2.4 cells at 1 × 10^5^ cells per well on 96-well round bottom plates were cultured in complete RPMI containing 10% FBS. The DC2.4 cells were stimulated with 10, 100, or 1000 μg/ml OVA adsorbed to 80 μg/ml Nano-11 or the combination adjuvant comprised of 80 μg/ml Nano-11 and 5 μg/ml ADU-S100 (NanoST) for 3 h at 37 °C with 5% CO_2_. Cells in media only or treated with 0.5 μg/ml OVA _257–264_ (SIINFEKL) served as negative and positive controls, respectively. The cells were centrifuged at 300 × *g* for 5 min and then washed three times with 200 μl of sterile PBS/0.1% BSA. Following fixation with 200 μl of PBS/0.2% paraformaldehyde for 15 min at 4 °C, cells were washed twice with complete RPMI. Each well was seeded with 1 × 10^5^ B3Z cells in complete RPMI containing 10% FBS for 16 h at 37 °C with 5% CO_2._ Following centrifugation at 300 × *g* for 5 min, cells were resuspended in 200 μl of lysis buffer containing 9 mm MgCl_2_, 0.125% NP40, and 0.15 mm chlorophenol red-β-D-galactopyranoside (CPRG, Sigma-Aldrich, St. Louis, MO). After 2, 4, 6, 8, 12, and 24 h, the conversion of CPRG by β -galactosidase was quantified by measuring the absorbance at 590 nm (OD 590) using a microplate reader (BioTek Instruments, Winooski, VT).

### Antigen-specific cytotoxic T cell assay in vivo

Splenocytes were isolated from naïve C57BL/6 J mice and treated with red blood cell ACK lysis buffer to create a single-cell suspension. A total of 2 × 10^7^ cells/ml were pulsed with 1 μm of SIINFEKL (pulsed) or left untreated (control) in HBSS and then incubated for 1 h at 37 °C with 5% CO_2_. The cells were centrifuged at 300 × *g* for 5 min, resuspended in complete RPMI 10% FBS, centrifuged once more, and resuspended in PBS/0.1% BSA. SIINFEKL-pulsed cells at a concentration of 5 × 10^6^ cells/ml were stained with the fluorescent dye CFSE at 2.5 μm (CFSE^hi^), whereas the control cells were stained with 0.25 μm (CFSE^low^), immediately vortexed, and incubated for 10 min at 37 °C with 5% CO_2_. A 5-min centrifugation at 300 × *g* was followed by a resuspension in complete RPMI 10% FBS, additional centrifugation, and a final resuspension in PBS. Untreated cells and cells pulsed with SIINFEKL were combined at a 1:1 ratio to provide a final cell concentration of 2 × 10^8^ cells/ml. One hundred μl (1 × 10^7^ of each cell population) was injected into the tail vein of mice that had undergone two intradermal vaccinations with PBS, OVA, Nano-11 + OVA, or NanoST + OVA, 21 days apart. The vaccinated mice were euthanized 4 h later, and the spleen and draining lymph node (superficial parotid) were isolated and processed into single-cell suspensions. Target lysis of CFSE-labeled cells was quantified by flow cytometry. The total events corresponding to both fluorescent intensities (CFSE^hi^ and CFSE^low^) were determined by flow cytometry. The percentage of lysis for each mouse was calculated as follows: % Lysis = 100-[(Total CFSE^hi^ cells/ Total CFSE^low^ cells) _vaccinated mice_ × 100 × (Total CFSE^low^ cells/ Total CFSE^hi^ cells) _PBS_].

### Preventive cancer vaccination

The efficacy of preventive cancer vaccines was investigated by two intradermal injections with OVA, Nano-11 + OVA, NanoST + OVA, or PBS with a 21-day interval. Eleven days following the second injection, mice were inoculated subcutaneously in the flank with 1.0 × 10^6^ B16-OVA, B16, or 2.5 × 10^6^ E.G7-OVA cells suspended in 100 μl of PBS. The tumor size was measured every other day, and the body weight was assessed to determine the body condition score using Ullman-Cullere’s scoring method^61^. Using a caliper, the length (maximum longitudinal dimension) and width (maximum transverse dimension) of the subcutaneous tumor were measured in order to determine the approximate volume. Tumor volume was calculated using the ellipsoidal formula: V = ½ × (Length × Width^2^)^57^. Each tumor was measured by three observers to account for interobserver variability. The tumor endpoint measurements were taken when the tumors reached a volume of 2,000 mm^3^, when the tumors became ulcerated, when the body condition scores dropped below 2, or when the mice became moribund. Mice without palpable tumors at 32 days after tumor inoculation were considered tumor-free and euthanized.

### Therapeutic cancer vaccination

To mimic therapeutic immunotherapy, mice were injected subcutaneously with 1.75 × 10^5^ B16-OVA or 2.5 × 10^6^ E.G7-OVA cells on day 0 to establish the tumor model. The B16-OVA-bearing mice were immunized with PBS, OVA, ADU-S100 + OVA, Nano-11 + OVA, or NanoST + OVA 5 days after tumor inoculation, followed by a second injection on day 12. A similar vaccination regimen was administered to E.G7-OVA-bearing mice at 1 and 21 days after tumor inoculation. The tumor endpoint measurements were recorded as described under the prophylactic cancer vaccine strategy. Mice without visible tumors were deemed tumor-free and euthanized 32 or 33 days post-tumor inoculation.

### Immune checkpoint blockade with therapeutic cancer vaccination

Mice were injected subcutaneously with 3.5 × 10^5^ B16-OVA or B16 cells on day 0 to establish the tumor model. The B16-OVA-bearing mice were intradermally immunized with NanoST ± OVA or B16 cell lysate on day 5, followed by a second vaccination on day 12. A second group of NanoST + B16 cell lysate or NanoST + OVA immunized mice were treated with anti-PD-1 (clone RMP1-14, Leinco) checkpoint inhibitors. Mice were treated with 200 μg of anti-PD-1 mAbs intraperitoneally 1, 3, and 6 days after the first vaccination. The tumor endpoint measurements were recorded as described under the prophylactic cancer vaccine strategy. Mice without visible tumors were deemed tumor-free and euthanized 32 days post-tumor inoculation.

### Depletion of CD4^+^ and CD8^+^ T cells

To evaluate the contribution of CD4^+^ and CD8^+^ T cell responses to prophylactic-mediated vaccine protection against B16-OVA and E.G7-OVA tumor formation, C57BL/6 J mice were vaccinated with Nano-11 + OVA or NanoST + OVA, and 21 days later the vaccination was repeated. Mice received 300 μg of anti-mouse CD4-depleting mAbs (clone GK1.5, Leinco, St. Louis, MO) or anti-mouse CD8α-depleting mAbs (clone 2.43, Leinco) 9 days after the second immunization through intraperitoneal injection, and continued to receive 100 μg of αCD4 and αCD8α depletion treatment for 3 weeks on every fourth day. Two days following the initial depletion treatment, the flanks of mice were injected subcutaneously with 1.0 × 10^6^ B16-OVA or 2.5 × 10^6^ E.G7-OVA cells. The tumor growth and survival of mice were monitored for 32 days post tumor inoculation.

### ELISA

OVA-specific IgG, IgG1, and IgG2c titers were measured by enzyme-linked immunosorbent assays (ELISA) on serum samples taken 10 days following the second vaccination, as previously reported^26^. In short, 96-well plates were coated with 1 μg/ml OVA overnight at 4 °C. After three washes with phosphate-buffered saline containing 0.05% TWEEN (PBST; Sigma-Aldrich), the plates were blocked at room temperature (RT) for 2 h with 200 μl PBST comprised of 1% BSA, followed by adding 100 μl of serially diluted serum samples to the wells in duplicate for 1 h at 37 °C. Wells were washed and incubated with 100 μl of peroxidase-conjugated goat anti-mouse IgG (1030-05), IgG1 (1073-05), or IgG2c (1079-05; SouthernBiotech, Birmingham, AL) for 1 h at 37 °C. Following a final wash, 100 μl of 3,3’,5,5’ tetramethylbenzidine substrate solution (Neogen, Lansing, MI) was added to all the wells and allowed to react in the dark for 10 min at RT. The reaction was stopped with the addition of 50 μl 2 m sulfuric acid, and absorbance at 450 nm (OD 450) was determined using the Synergy HT microplate reader (BioTek, Winooski, VT). End-point titers for OVA-specific antibodies were determined when the dilution at which OD 450 nm approached 0.2. The final OD values were obtained by subtracting the average value of three blank samples from the OD of the experimental test samples.

### ELISpot

The ELISpot assay was performed to quantify the number of OVA-specific antibody-secreting cells (ASCs) in bone marrow cells extracted from the tibias and femurs of mice 10 days after the second vaccination, as previously described^26^. Briefly, MultiScreen IP filter plates (MAIPS4510; Sigma-Aldrich) were prepared by activating the filter membranes with 20 μl/well of 35% ethanol for 30 sec and then washing the plates with 300 μl/well of sterile PBS three times. The wells were coated overnight at 4 ˚C with 10 μg/ml of OVA (100 μl/well) in sterile PBS. Wells were washed with sterile PBS and then blocked with complete RPMI (RPMI 1640 supplemented with 2 mm L-glutamine, 55 μm beta-mercaptoethanol, 1 × non-essential amino acids, 10 mm HEPES, 100 U/ml penicillin, 100 μg/ml streptomycin, and 0.25 μg/ml amphotericin) with 10% FBS for 2 h at 37 °C, followed by incubating the serially diluted single-cell suspensions of bone marrow cells in duplicate at 37 °C with 5% CO_2_ for 24 h. The plates were then washed five times with PBST and incubated at RT for 2 h with biotin-labeled anti-mouse IgG (SouthernBiotech) in 1% FBS-containing PBST. This was followed by five washes, and then the plates were incubated with avidin-HRP conjugate (Thermo Fisher Scientific) in 1% FBS-containing PBST for 1 h at 37 °C. To initiate enzymatic activity, wells were exposed to 3-amino-9-ethylcarbazole (Sigma-Aldrich) treatment and protected from light for 10 min, followed by 15 washes with deionized water. The membrane tray was protected from light and dried at RT before the red-colored spots were quantified utilizing an ELISPOT reader (AID Diagnostika, Strassberg, Germany).

### Flow cytometry

Single-cell suspensions were prepared from isolated splenocytes or lymph nodes and washed with Cell Staining Buffer (CSB; BioLegend, San Diego, CA). The staining protocols were conducted in accordance with the manufacturer’s instructions, and unless otherwise specified, all mAbs were obtained from BioLegend. Live, viable cells were treated for 30 min at 4 °C with an anti-mouse CD16/32 (clone 93) antibody. A total of 2 × 10^6^ splenocytes were labeled with anti-mouse mAbs against CD3ε (clone 145-2C11), CD4 (clone GK1.5), CD8α (clone 53-6.7), CD185/CXCR5 (clone L138D7), CD279/PD-1 (clone 29 F.1A12), GL-7 (clone GL7), CD45R/B220 (clone RA3-6B2), and CD95/FAS (clone SA367H8) in CSB for 45 min at 4 °C. Identification of SIINFEKL-specific CD8^+^ T cells was achieved by utilizing a H-2K(b) chicken OVA _257–264_ SIINFEKL tetramer (RRID: AB_3068342) donated from the NIH Tetramer Core Facility at Emory University (Atlanta, GA). A total of 1 × 10^6^ splenocytes were utilized for tetramer staining. In order to perform intracellular cytokine staining (ICS), splenocytes were first cultured in complete RPMI supplemented with 10% FBS and 25 μg/ml OVA for 24 h at 37 °C with 5% CO_2_. Thereafter, the cells were stimulated at 37 °C with 5% CO_2_ for 6 h with PMA, ionomycin, and monensin in complete RPMI supplemented with 10% FBS. Subsequently, the splenocytes were labeled with mAbs following the same method as described above and then permeabilized using Perm Wash Buffer (BioLegend) in preparation for ICS with IFN-γ (clone XMG1.2) and IL-17A (clone TC11-18H10.1). The cells were washed with CSB prior to getting fixed using a 4% paraformaldehyde fixation buffer (BioLegend). Human THP-1 cells were labeled with anti-human CD11b (clone ICRF44), anti-human CD80 (clone 2D10), and anti-human CD86 (clone BU63) mAbs after 30 min of treatment with human TruStain FcX (BioLegend) at 4 °C. Flow cytometry was conducted using an Attune NxT flow cytometer (Invitrogen, Waltham, MA), and the data was analyzed using FlowJo software (FlowJo, Ashland, OR).

### Statistical analysis

To identify statistically significant differences between experimental groups, a one- or two-way analysis of variance (ANOVA) test was conducted, followed by Tukey’s or Sidak’s multiple comparison test. The percentage of tumor-free mice and survival rate were determined using the log-rank (Mantel-Cox) curve comparison test. All statistics were performed using GraphPad Prism (version 9.2, San Diego, CA). The results are presented as mean ± SEM. A value of *p* < 0.05 indicated statistical significance (*p < 0.05, **p < 0.01, ***p < 0.001, and ****p < 0.0001).

### Supplementary information

Supplementary Information

## Data Availability

This study did not generate any data requiring mandatory deposition. Additional data from this study will be provided upon reasonable request to jfhernan@purdue.edu or hogenesc@purdue.edu. The data that support the findings of this study are available from the corresponding author upon reasonable request.
