# Supplementary material for: Intradermal vaccination with a phytoglycogen nanoparticle and STING agonist induces cytotoxic T lymphocyte-mediated antitumor immunity
Source: NPJ Vaccines. 2024 Aug 17;9:149. doi: 10.1038/s41541-024-00943-8 (PMC11329758; doi:10.1038/s41541-024-00943-8)
Supplement: Supplementary file 1 — Supplementary Information [file 41541_2024_943_MOESM1_ESM.pdf]

Formulation	Particle size (nm)	PDI	Zeta potential (mV)
OVA	2.8 ± 0.0	0.3 ± 0.0	-1.8 ± 1.3
B16 cell lysate	69.4 ± 13.1	0.9 ± 0.1	-19.5 ± 1.3
Nano-11	68.0 ± 1.0	0.2 ± 0.0	24.9 ± 1.0
Nano-11 + OVA	72.5 ± 0.7	0.2 ± 0.0	29.6 ± 0.7
Nano-11 + B16 cell lysate	158.1 ± 5.8	0.7 ± 0.0	34.8 ± 0.5
NanoST	68.5 ± 0.6	0.1 ± 0.0	20.9 ± 0.7
NanoST + OVA	72.8 ± 1.0	0.2 ± 0.0	25.5 ± 1.7
NanoST + B16 cell lysate	149.4 ± 2.8	0.8 ± 0.0	32.8 ± 0.9

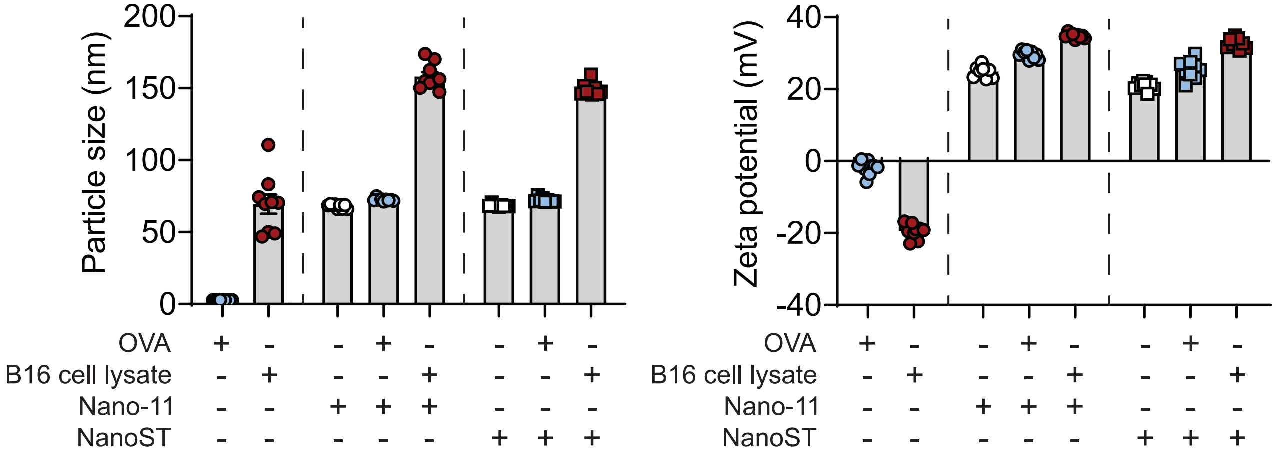

Supplementary Figure 1. Physical characterization of Nano-11 and NanoST-based vaccine formulations. The vaccines were formulated by adsorbing ovalbumin (OVA) or B16 cell lysate to Nano-11 or NanoST. The Z-average hydrodynamic diameter (particle size) and the particle surface charge (zeta-potential) was determined with a zetasizer. The polydispersity index (PDI) represents variance within the vaccine formulations. Three independent vaccines were formulated for each group to obtain the measurements.
